# Supplementary material for: Causal Relationship between Aging and Anorexia Nervosa: A White-Matter-Microstructure-Mediated Mendelian Randomization Analysis
Source: Biomedicines. 2024 Aug 16;12(8):1874. doi: 10.3390/biomedicines12081874 (PMC11351342; doi:10.3390/biomedicines12081874)
Supplement: Supplementary file 1 [file biomedicines-12-01874-s001.zip › Supplementary Figure/Figure S9/Leaveoneout plot of MR analysis for ubm-b-1952 to AN.pdf]

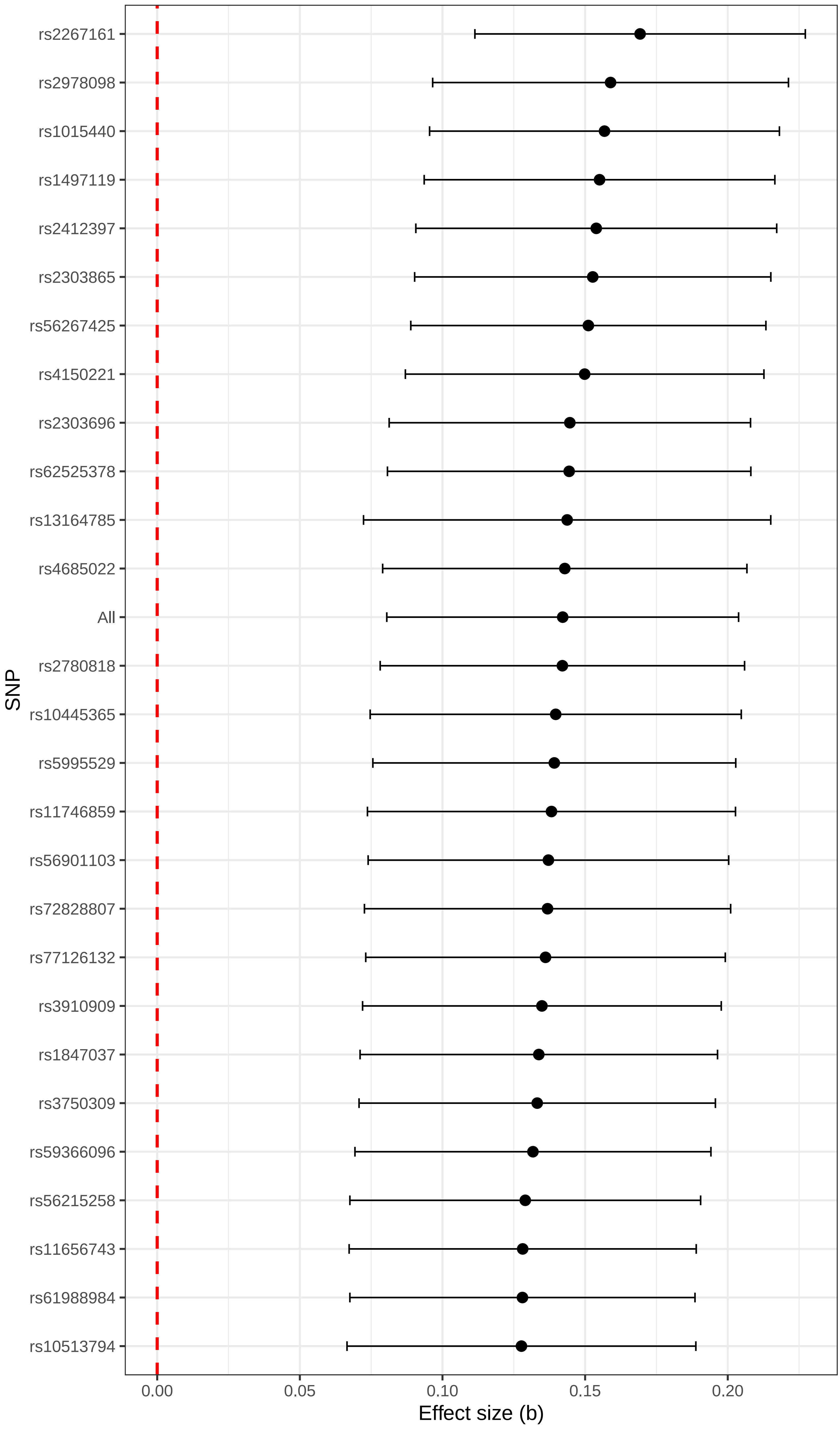
rs2267161
rs2978098
rs1015440
rs1497119
rs2412397
rs2303865
rs56267425
rs4150221
rs2303696
rs62525378
rs13164785
rs4685022
All
rs2780818
rs10445365
rs5995529
rs11746859
rs56901103
rs72828807
rs77126132
rs3910909
rs1847037
rs3750309
rs59366096
rs56215258
rs11656743
rs61988984
rs10513794
SNP
0.00
0.05
0.10
0.15
0.20
Effect size (b)
